# Inhibition of Microglia-Derived Oxidative Stress by Ciliary Neurotrophic Factor Protects Dopamine Neurons In Vivo from MPP^+^ Neurotoxicity

**DOI:** 10.3390/ijms19113543

**Published:** 2018-11-10

**Authors:** Jeong Yeob Baek, Jae Yeong Jeong, Kyoung In Kim, So-Yoon Won, Young Cheul Chung, Jin Han Nam, Eun Ju Cho, Tae-Beom Ahn, Eugene Bok, Won-Ho Shin, Byung Kwan Jin

**Affiliations:** 1 Department of Neuroscience, Graduate School, Kyung Hee University, Seoul 02447, Korea; yeobe88@khu.ac.kr (J.Y.B.); jyoung0229@khu.ac.kr (J.Y.J.); basketis@khu.ac.kr (K.I.K.); 2 Department of Biochemistry and Signaling Disorder Research Center, College of Medicine, Chungbuk National University, Cheongju 28644, Korea; sywon@chungbuk.ac.kr; 3 Department of Biochemistry & Molecular Biology, School of Medicine, Kyung Hee University, Seoul 02447, Korea; ychung01@khu.ac.kr (Y.C.C.); namkoon02@gmail.com (J.H.N.); bioneun00@yonsei.ac.kr (E.J.C.); 4 Department of Neurology, School of Medicine, Kyung Hee University, Seoul 02447, Korea; doctorbrain@empal.com; 5 Department of Predictive Toxicology, Korea Institute of Toxicology, Daejeon 34114, Korea; luckyars@naver.com

**Keywords:** Parkinson’s disease, Microglia, Ciliary neurotrophic factor, Ciliary neurotrophic factor receptor, oxidative stress

## Abstract

We demonstrated that capsaicin (CAP), an agonist of transient receptor potential vanilloid subtype 1 (TRPV1), inhibits microglia activation and microglia-derived oxidative stress in the substantia nigra (SN) of MPP^+^-lesioned rat. However, the detailed mechanisms how microglia-derived oxidative stress is regulated by CAP remain to be determined. Here we report that ciliary neurotrophic factor (CNTF) endogenously produced by CAP-activated astrocytes through TRPV1, but not microglia, inhibits microglial activation and microglia-derived oxidative stress, as assessed by OX-6 and OX-42 immunostaining and hydroethidine staining, respectively, resulting in neuroprotection. The significant increase in levels of CNTF receptor alpha (CNTFRα) expression was evident on microglia in the MPP^+^-lesioned rat SN and the observed beneficial effects of CNTF was abolished by treatment with CNTF receptor neutralizing antibody. It is therefore likely that CNTF can exert its effect via CNTFRα on microglia, which rescues dopamine neurons in the SN of MPP^+^-lesioned rats and ameliorates amphetamine-induced rotations. Immunohistochemical analysis revealed also a significantly increased expression of CNTFRα on microglia in the SN from human Parkinson’s disease patients compared with age-matched controls, indicating that these findings may have relevance to the disease. These data suggest that CNTF originated from TRPV1 activated astrocytes may be beneficial to treat neurodegenerative disease associated with neuro-inflammation such as Parkinson’s disease.

## 1. Introduction

Microglia are the resident macrophages in the brain and spinal cord [1]. Many studies have demonstrated that activated microglia play a pivotal role in the cause and progression of Parkinson’s disease (PD) characterized by the loss of dopamine (DA) neurons in the substantia nigra (SN) and motor dysfunctions [2,3,4,5]. Activated microglia exhibit large cell bodies with short, thick or no processes and exert neurotoxic effects by producing and releasing reactive oxygen species (ROS), inflammatory cytokines, which consequently triggers oxidative stress and severe inflammation. Microglia-derived oxidative stress and inflammation are involved in degeneration of DA neurons in the SN of patients with PD [3,4,5,6,7,8] and in the 1-methyl-4-phenylpyridinium (MPP^+^)-lesioned rats [9].

Transient receptor potential vanilloid subtype 1 (TRPV1), the capsaicin (CAP) receptor, is involved in pain perception and is highly expressed in sensory neurons [10]. TRPV1 is also present in the brain [11,12] including DA neurons and astrocytes in the SN of intact and MPP^+^-lesioned rat and patients with PD [13]. We recently showed that TRPV1 activation by CAP contributes to DA neuronal survival by inhibiting microglia-derived oxidative stress in the MPP^+^-lesioned SN [9]. The study also demonstrated that pharmacological inhibition of TRPV1 activation by the TRPV1 antagonist capsazepine attenuated CAP-induced neuro-protection and inhibition of microglia-derived ROS production, indicating TRPV1-dependent effects of CAP. CAP has also been found to decrease intracellular ROS levels in myoblasts [14] and apoptosis in hippocampal neurons [15], collectively suggesting that CAP-induced TRPV1 activation may regulate oxidative stress. By contrast, several studies showed TRPV1-independent effects of CAP on glial cells. Pharmacological blockade by capsazepine or genetic deficiency of TRPV1 did not influence CAP-induced suppression of the production of prostaglandin 2 (PGE_2_), inducible nitric oxide synthase (iNOS) and cyclooxygenase-2 (COX-2), and free radical formation in activated microglia and macrophages [16,17,18].

Ciliary neurotrophic factor (CNTF) is expressed in astrocytes [19,20,21] and microglia [22,23]. CNTF was found to stimulate astrocytes to secret fibroblast growth factor-2 [24] and rat microglia to secret glial cell line-derived neurotrophic factor [25], which suggest that CNTF exerts effects on glial cells to promote motor neuron survival [26,27,28]. CNTF attenuated microglial activation in optic nerve lesion [29] and increased motor neuron survival by reducing expression of microglial COX-2 related to oxidative stress [25]. CNTF exhibits biological functions through its own receptor, which consists of three subunits: CNTF receptor alpha (CNTFRα), Glycoprotein 130, and Leukemia inhibitory factor receptor [30]. CNTFRα is expressed on astrocytes [20,31,32] and microglia [25,33]. Activation of CNTFRα by CNTF enhanced microglial PGE_2_ secretion and COX-2 protein expression in cultured mouse microglia [33]. We have recently demonstrated expression of CNTFRα in DA neurons in the SN of intact and MPP^+^-lesioned rats and of patients with PD [13]. Endogenous CNTF produced by TRPV1 activation on astrocytes acts through CNTFRα on DA neurons in the SN in vivo of MPP^+^-lesioned rats, resulting in neuro-protection and behavioral recovery [13]. CNTF also regulates microglia activation [25,33,34,35,36]. Here we report that endogenous CNTF originating from astrocytic TRPV1 activation by CAP inhibits microglial activation and microglia-derived oxidative stress in vivo by acting through CNTFRα on microglia, in consequence, rescuing DA neurons and improving motor recovery in the MPP^+^-lesioned rat model of PD. The present data delve deeper into understanding the novel neuroprotective mechanism by which CAP inhibits microglia-derived ROS production and oxidative stress via astrocytic TRPV1-derived CNTF, which is acting via CNTFRα on microglia.

## 2. Results

### 2.1. TRPV1 Activation by Capsaicin Prevents Degeneration of DA Neurons and Inhibits Microglial Activation In Vivo in MPP^+^-Lesioned Rat

**Figure 1:**
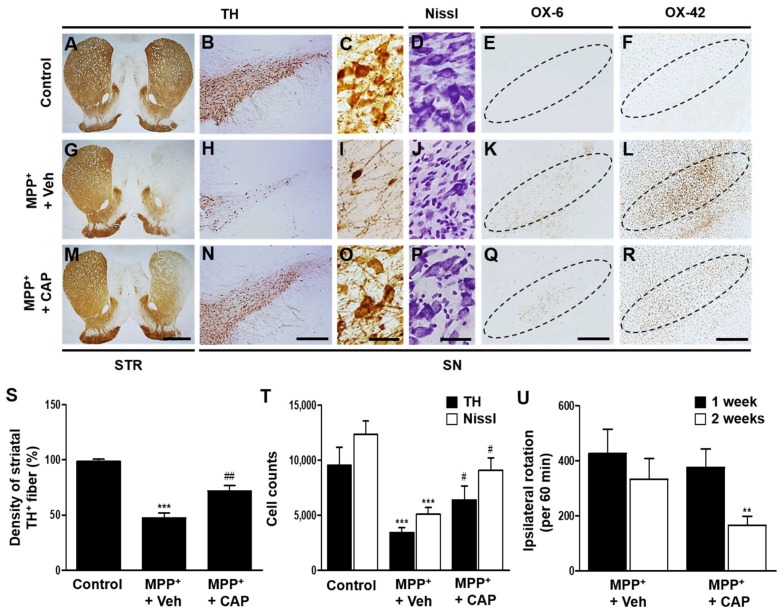
TRPV1 activation by capsaicin produces DA neuroprotection and reduces microglial activation in vivo in MPP^+^-lesioned rat. MPP^+^ was unilaterally injected into the rat medial forebrain bundle (MFB) followed by intraperitoneal (i.p.) treatment of capsaicin (CAP: 1 mg/kg) or vehicle (Veh) at 8 days post MPP^+^ injection and a continuous single injection per day for 7 days. Rats were transcardially perfused after the last amphetamine-induced rotation experiment. (**A**–**R**) Photomicrographs of TH^+^ fibers (**A**,**G**,**M**) in the striatum (STR), and TH^+^ (**B**,**C**,**H**,**I**,**N**,**O**), Nissl^+^ (**D**,**J**,**P**), OX-6^+^ (**E**,**K**,**Q**) and OX-42^+^ (**F**,**L**,**R**) cells in the substantia nigra (SN). (**S**) Optical density of striatal TH^+^ fibers. One way ANOVA [F(2,19) = 26.92, *p* < 0.0001] and Newman-Keuls analysis, *** *p* < 0.001, significantly different from control, ^##^ *p* < 0.01, significantly different from MPP^+^ + Veh. (**T**) Number of TH^+^ or Nissl^+^ cells in the SN. One way ANOVA [F_TH_(2,17) = 10.29, *p* = 0.0012; F_Nissl_(2,21) = 12.63, *p* = 0.0003] and Newman-Keuls analysis, *** *p* < 0.001, significantly different from control; ^#^ *p* < 0.05, significantly different from MPP^+^ + Veh. (**U**) Cumulative amphetamine-induced ipsilateral rotations. Student *t*-Test analysis, ** *p* < 0.01 (*t* = 2.785, df = 30), significantly different from 1 week. Dotted lines indicate SN. Scale bars: 1 mm (**A**,**G**,**M**), 400 μm (**B**,**E**,**F**,**H**,**K**,**L**,**N**,**Q**,**R**), 40 μm (**C**,**D**,**I**,**J**,**O**,**P**). Mean ± S.E.M; (**S**) *n* = 6 to 9; (**T**) *n* = 4 to 8; (**U**) *n* = 12 to 16.

Rats unilaterally received MPP^+^ or PBS as a control in the medial forebrain bundle (MFB). At 2 weeks after MPP^+^ injection, analysis by TH immunohistochemistry revealed 51% reduction in density of TH^+^ fibers in the striatum (Figure 1A,G,S), and loss of TH^+^ cells by 67% and Nissl^+^ cells by 58% in the SN (Figure 1B–D,H–J,T), concomitant with amphetamine-induced rotations (Figure 1U), compared to PBS control. In MPP^+^ lesioned rat, the TRPV1 agonist, CAP increased the density of striatal TH^+^ fiber by 53% (Figure 1M,S), and the number of TH^+^ cells by 58% and Nissl^+^ cells by 46% in the SN, respectively (Figure 1N–P,T) and ameliorated amphetamine-induced rotations (Figure 1U), compared to vehicle-treated control.

Given the neurotoxicity of activated microglia on DA neurons in the MPP^+^-lesioned SN [9], we hypothesized that MPP^+^ neurotoxicity is in parallel with microglial activation in vivo. We found that MPP^+^-induced degeneration of DA neurons was accompanied by microglial activation as visualized by OX-6 (marker for activated microglia) and OX-42 (general marker for microglia) immunostaining in the SN (Figure 1K,L). CAP treatment decreased the immunoreactivity of OX-6^+^ and OX-42^+^ cells in the SN at 2 weeks post MPP^+^ injection compared to vehicle-treated MPP^+^-lesioned SN (Figure 1Q,R).

### 2.2. Astrocytic TRPV1 Knockdown by shRNA Alters DA Neuronal Survival and Microglial Activation In Vivo in MPP^+^-Lesioned Rat

As astrocytic TRPV1 activation by CAP contributes to DA neuronal survival in vivo [13], we wondered whether it could exert neuroprotection by regulating microglial activation. To test this hypothesis, astrocytic TRPV1 function was selectively and efficiently inhibited by a lentivirus carrying a small hairpin-forming interference RNA (shRNA) targeted against TRPV1 (shTRPV1) [13] (also see Section 4.10). The virus also contained DNA encoding a fluorescent marker, EGFP, which permitted visualization of the location and amount of viral infection.

**Figure 2:**
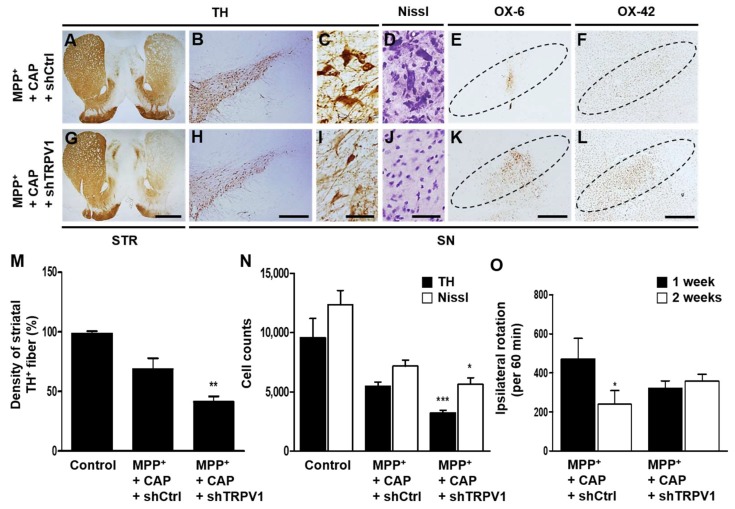
Knockdown of astrocytic TRPV1 induces degeneration on DA neurons and increase of microglial activation in vivo in MPP^+^-lesioned rat. MPP^+^ was unilaterally injected into the rat MFB followed by injection of shCtrl (control) or shTRPV1 into the SN. All rats i.p. received CAP at 8 days post MPP^+^ injection and a continuous single injection per day for 7 days. Rats were transcardially perfused after the last amphetamine-induced rotation experiment. (**A**–**L**) Photomicrographs of TH^+^ fibers (**A**,**G**) in the STR, and TH^+^ (**B**,**C**,**H**,**I**), Nissl^+^ (**D**,**J**), OX-6^+^ (**E**,**K**) and OX-42^+^ (**F**,**L**) cells in the SN. (**M**) Optical density of striatal TH^+^ fibers. Student *t*-Test analysis, ** *p* < 0.01 (*t* = 2.782, df = 11), significantly different from MPP^+^ + CAP + shCtrl. (**N**) Number of TH^+^ or Nissl^+^ cells in the SN. Student *t*-Test analysis, * *p* < 0.05 (*t* = 1.968, df = 10), *** *p* < 0.001 (*t* = 5.792, df = 10), significantly different from MPP^+^ + CAP + shCtrl. (**O**) Cumulative amphetamine-induced ipsilateral rotations. Student *t*-Test analysis, * *p* < 0.05 (*t* = 1.837, df = 10), significantly different from 1 week. Dotted lines indicate SN. Scale bars: 1 mm (**A**,**G**), 400 μm (**B**,**E**,**F**,**H**,**K**,**L**), 40 μm (**C**,**D**,**I**,**J**). Mean ± S.E.M.; (**M**) *n* = 6 to 7; (**N**) *n* = 5 to 7; (**O**) *n* = 6 to 8.

To examine the selectivity and efficacy of shRNA in vivo, we stereotaxically injected MPP^+^ and control scrambled shRNA (shCtrl) in the SN (see Section 4.3). We observed EGFP expression of shCtrl mainly within astrocytes 7 days post MPP^+^ injection (Figure S1A,B). shTRPV1 selectively and efficiently inhibited TRPV1 expression primarily within astrocytes in the SN of MPP^+^-lesioned rat (Figure S1C,D). To evaluate the role of astrocytic TRPV1 on DA neurons in the SN, control scrambled shRNA (shCtrl) or shTRPV1 was ipsilaterally injected into the SN immediately after the unilateral MFB injection of MPP^+^. In the CAP-treated MPP^+^-lesioned rats, shTRPV1 decreased the density of TH^+^ fibers by 54% in the striatum (Figure 2A,G,M), and the numbers of TH^+^ cells by 69% and Nissl^+^ cells by 76% in the SN (Figure 2B–D,H–J,N) as well as reducing functional recovery (Figure 2O) compared to shCtrl.

In parallel with aggravation of DA neuronal loss and behavioral deficits by shTRPV1, astrocytic TRPV1 deficiency significantly increased immunostaining intensity of OX-6^+^ (Figure 2K) and OX-42^+^ (Figure 2L) cells in the SN compared with shCtrl-treated control rats. Collectively, these results indicate that inhibition of microglial activation by astrocytic TRPV1 activation might contribute to neuroprotection and motor recovery.

### 2.3. CAP Treatment Is Unable to Change Basal Levels of CNTF on Microglia in the SN In Vivo of MPP^+^-Lesioned Rat

**Figure 3:**
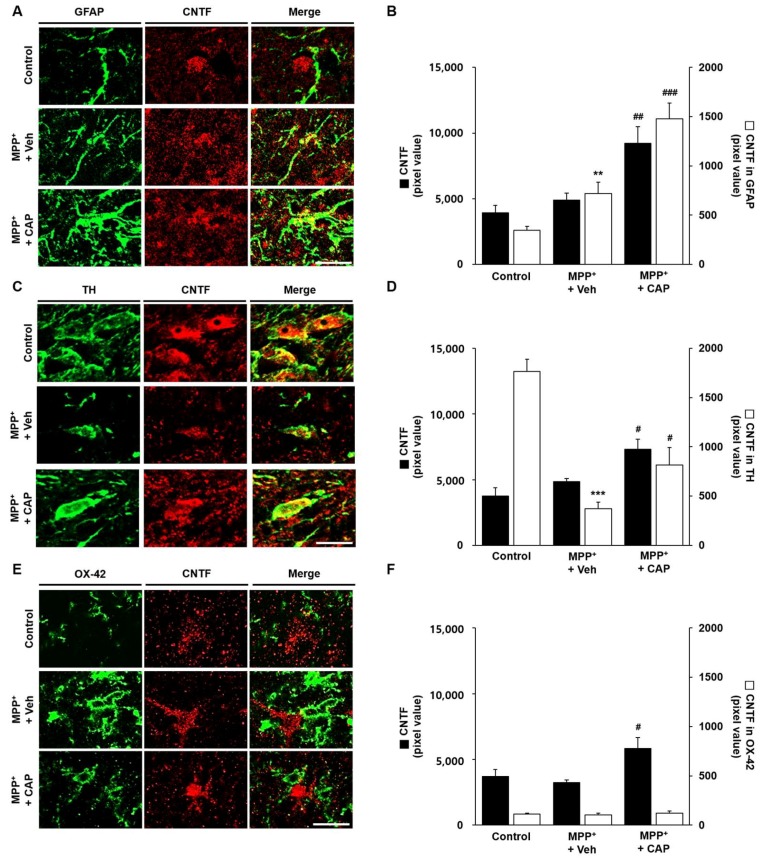
CNTF expression in the SN in vivo of MPP^+^-lesioned rat. MPP^+^ was unilaterally injected into the rat MFB followed by i.p. treatment of CAP (1 mg/kg) or Veh at 8 days post MPP^+^ injection and a continuous single injection per day for 7 days. (**A**) Fluorescence images of CNTF (red; **A**) or GFAP^+^ cells (green; **A**) and both images are merged in the rat SN at 2 weeks post MPP^+^ injection. (**B**) Quantification of CNTF or CNTF expression in GFAP^+^ cells. One way ANOVA [F_CNTF_(2,9) = 12.42, *p* = 0.0026, F_CNTF in GFAP_(2,11) = 37.24, *p* < 0.0001] and Newman–Keuls analysis, ** *p* < 0.01, significantly different from control. ^##^ *p* < 0.01, ^###^ *p* < 0.001, significantly different from MPP^+^ + Veh. (**C**) Fluorescence images of CNTF (red; **C**) or TH^+^ cells (green; **C**) and both images are merged in the rat SN at 2 weeks post MPP^+^ injection. (**D**) Quantification of CNTF or CNTF expression in TH^+^ cells. One way ANOVA [F_CNTF_(2,15) = 8.675, *p* = 0.0031, F_CNTF in TH_(2,14) = 27.53, *p* < 0.0001] and Newman–Keuls analysis, *** *p* < 0.001, significantly different from control. ^#^ *p* < 0.05, significantly different from MPP^+^ + Veh. (**E**) Fluorescence images of CNTF (red; **E**) or OX-42^+^ cells (green; **E**) and both images are merged in the rat SN at 2 weeks post MPP^+^ injection. (**F**) Quantification of CNTF or CNTF expression in OX-42^+^ cells. One way ANOVA [F_CNTF_(2,10) = 5.246, *p* = 0.0277] and Newman–Keuls analysis, ^#^ *p* < 0.05, significantly different from MPP^+^ + Veh. Scale bars; 20 μm. Mean ± S.E.M.; (**B**) *n* = 3 to 7; (**D**) *n* = 5 to 7; (**F**) *n* = 4 to 6.

As CNTF has neuro-protective properties [37], and inhibits microglial activation [29] and/or microglia-derived oxidative stress [25], CNTF expression was assessed in the absence or presence of CAP in the SN in vivo of MPP^+^-lesioned rat. At 2 weeks post MPP^+^ injection, analysis by immunohistochemical staining showed that CNTF expression in GFAP^+^ astrocytes was significantly higher in the rat SN compared to control (Figure 3A,B). CNTF expression in TH^+^ neurons was significantly reduced (Figure 3C,D), whereas basal level of CNTF expression in OX-42^+^ microglia was unchanged (Figure 3E,F) compare to control. In CAP-treated MPP^+^-lesioned SN, CNTF expression in both GFAP^+^ astrocytes (Figure 3A,B) and TH^+^ neurons (Figure 3C,D) was significantly increased, whereas CNTF expression in OX-42^+^ microglia was relatively unchanged (Figure 3E,F) compared to vehicle-treated MPP^+^-lesioned SN. Collectively, the results indicate that CAP induces expression of CNTF originated from astrocytes, but not microglia. Knockdown of astrocytic TRPV1 reduced CNTF expression in the absence or presence of CAP in the SN of MPP^+^-lesioned rat (data not shown), supporting our recent findings of astrocytic TRPV1 activation-derived CNTF expression [13].

### 2.4. CNTFRα Neutralization Alters Neuroprotection and Microglial Activation in the SN In Vivo in MPP^+^-Lesioned Rat

**Figure 4:**
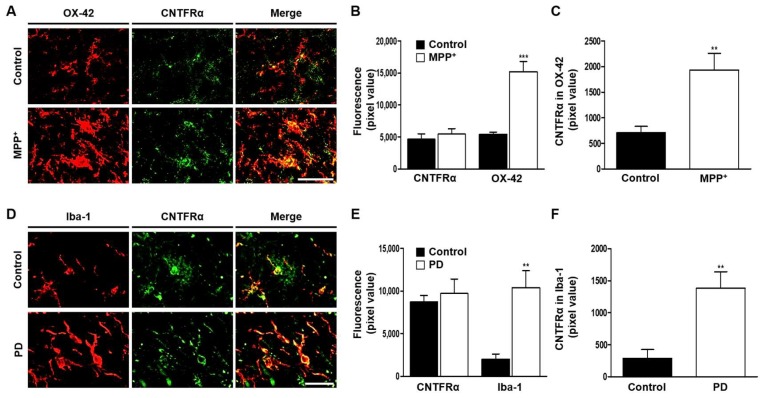
CNTFRα expression on microglia in the SN of the MPP^+^-lesioned rat and human PD brain. (**A**–**C**) MPP^+^ was unilaterally injected into the rat MFB and tissues were prepared for immunohistochemical analysis at 1 week post MPP^+^ injection. (**A**) Fluorescence images of CNTFRα (green) or OX-42 (red) and both images are merged in the rat SN 1 week post MPP^+^ injection into rat MFB. Quantification of CNTFRα expression and OX-42^+^ cells (B), and CNTFRα expression in OX-42^+^ cells (**C**). Student *t*-Test analysis, ** *p* < 0.01 (*t*_OX-42_ = 3.513, df = 8), *** *p* < 0.001 (*t*_CNTFR__α in OX-42_ = 6.115, df = 8) significantly different from control. (**D**–**F**) CNTFRα expression in the SN of human PD brain. (**D**) Fluorescence images of CNTFRα (green) and Iba-1^+^ microglia (red) and both images are merged. Quantification of CNTFRα or Iba-1^+^ microglia (**E**), and CNTFRα expression in Iba-1^+^ microglia (**F**). Student *t*-Test analysis, ** *p* < 0.01 (*t*_Iba-1_ = 3.971, df = 4), ** *p* < 0.01 (*t*_CNTFR__α in Iba-1_ = 3.785, df = 4), significantly different from control. Scale bars; 50 μm (**A**), 20 μm (**D**). Mean ± S.E.M; (**B**,**C**) *n* = 5; (**E**,**F**) *n* = 3.

As CNTF primarily functions via CNTFRα [30], we examined if CNTF could exert its effect on microglia through CNTFRα expressed on microglia. Immunohistochemical analysis showed a significant increase in the level of CNTFRα expression in OX-42^+^ cells in the SN of MPP^+^-lesioned rats, compared with control (Figure 4A–C). CNTFRα in Iba-1^+^ microglia was also significantly expressed in the SN of PD brain compared to age-matched human control brain (Figure 4D–F; Table 1), suggesting the possible relevance to PD.

**Figure 5:**
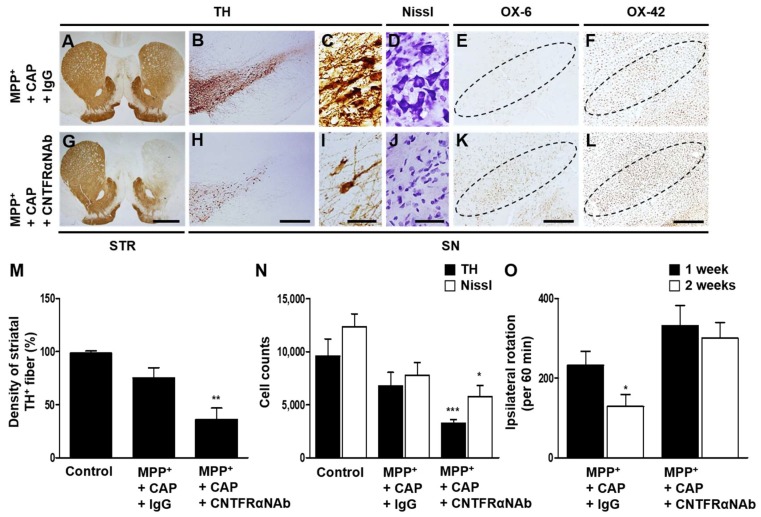
Effects of CNTFRα on neuro-protection and microglial activation in the SN in vivo in MPP^+^-lesioned rat. MPP^+^ was unilaterally injected into the rat MFB followed by intranigral injection of CNTFRα neutralizing antibody (CNTFRαNAb) or IgG (control) at 1 week post MPP^+^ injection. All rats i.p. received CAP at 8 days post MPP^+^ and a continuous single injection per day for 7 days. Rats were transcardially perfused after the last amphetamine-induced rotation experiment. (**A**–**L**) Photomicrographs of TH^+^ fibers (**A**,**G**) in the STR, and TH^+^ (**B**,**C**,**H**,**I**), Nissl^+^ (**D**,**J**), OX-6^+^ (**E**,**K**) and OX-42^+^ (**F**,**L**) cells in the SN. (**M**) Optical density of striatal TH^+^ fibers. Student *t*-Test analysis, ** *p* < 0.01 (*t* = 2.720, df = 15), significantly different from MPP^+^ + CAP + IgG. (**N**) Number of TH^+^ or Nissl^+^ cells in the SN. Student *t*-Test analysis, * *p* < 0.05 (*t* = 1.883, df = 10), *** *p* < 0.001 (*t* = 4.355, df = 10), significantly different from MPP^+^ + CAP + IgG. (**O**) Cumulative amphetamine-induced ipsilateral rotations. Student *t*-Test analysis, * *p* < 0.05 (*t* = 2.239, df = 24), significantly different from 1 week. Dotted lines indicate SN. Scale bars: 1 mm (**A**,**G**), 400 μm (**B**,**E**,**F**,**H**,**K**,**L**), 40 μm (**C**,**D**,**I**,**J**). Mean ± S.E.M; (**M**) *n* = 8 to 9; (**N**) *n* = 5 to 7; (**O**) *n* = 10 to 13.

Accordingly, we tested the effects of CNTFRα on microglial activation. CNTFRα neutralizing antibody (CNTFRαNAb) was unilaterally injected to block CNTF actions in the ipsilateral rat SN at 1 week after unilateral MFB injection of MPP^+^. Non-specific IgG was used as a control. CAP-induced neuro-protection and motor recovery were diminished as assessed by the density of TH^+^ fibers by 38% in the striatum (Figure 5A,G,M), and the numbers of TH^+^ cells by 39% and Nissl^+^ cells by 63% in the SN (Figure 5B–D,H–J,N) and amphetamine-induced rotations (Figure 5O) in MPP^+^-lesioned rats treated with CNTFRαNAb compared with non-specific IgG control rats. In parallel, in the CAP-treated MPP^+^-lesioned rats, CNTFRαNAb was found to increase immunostaining intensity of OX-6^+^ (Figure 5K) and OX-42^+^ (Figure 5L) cells in the SN, compared with non-specific IgG-treated respective control rats (Figure 5E,F). Taken together, these results indicate that CAP-induced astrocytic TRPV1-derived CNTF attenuates microglial activation through microglial CNTFRα.

### 2.5. CNTF Derived from TRPV1 Activated Astrocytes Inhibits Microglial ROS Production in the SN In Vivo in MPP^+^-Lesioned Rat

**Figure 6:**
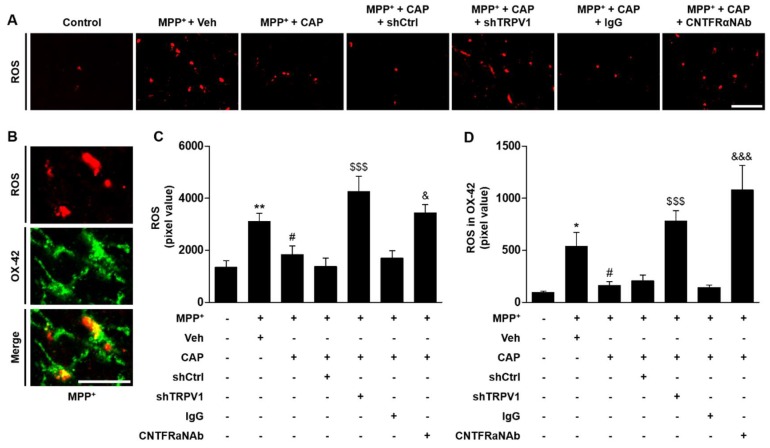
CNTF originated from TRPV1 activated astrocytes inhibits microglia-derived oxidative stress in the SN in vivo in MPP^+^-lesioned rat. At 2 weeks post MPP^+^ injection with indicated treatment, hydroethidine was administered through tail vein. After 45 min, the rats were transcardially perfused and immunohistochemical analyzed. (**A**) Fluorescence images of ROS (red, ethidium fluorescence) in the SN. (**B**) Fluorescence images of ROS (red) or OX-42 (green) and both images are merged (yellow) in the rat SN at 1 week post MPP^+^ injection. Quantification of ROS expression (**C**), and ROS expression in OX-42^+^ cells (**D**). One way ANOVA [F_ROS_(6,43) = 9.608, *p* < 0.0001; F_ROS in OX-42_(6,38) = 13.67, *p* < 0.0001] and Newman–Keuls analysis, * *p* < 0.05, ** *p* < 0.01, significantly different from control; ^#^ *p* < 0.05, significantly different from MPP^+^ + Veh; ^$$$^ *p* < 0.001, significantly MPP^+^ + CAP + shCtrl; ^&^ *p* < 0.05, ^&&&^ *p* < 0.001, significantly different from MPP^+^ + CAP + IgG. Scale bars: 40 μm (**A**), 20 μm (**B**), Mean ± S.E.M.; (**C**) *n* = 5 to 9; (**D**) *n* = 5 to 8.

Activated microglia generates ROS, which may impose oxidative stress on DA neurons in the SN of PD animal models [38,39] and in the SN of patients with PD [3,4,7], leading to DA neuronal cell death. As CNTF seems to exert its neuro-protection through inhibiting microglial activation, we hypothesized that CNTF could inhibit activated microglia-derived ROS production via CNTFRα on microglia. To test this, hydro-ethidine histochemistry was performed for in situ visualization of O_2_^−^ production. MPP^+^ significantly increased O_2_^−^ production (visualized as the fluorescent product of oxidized hydroethidine, i.e., ethidium accumulation) compared to control (Figure 6A). Microglia-derived O_2_^−^ production was then visualized using double immunofluorescence staining, which showed co-localization of hydroethidine staining within OX-42^+^ cells (Figure 6B). In the CAP-treated MPP^+^-lesioned rats, shTRPV1 significantly increased O_2_^−^ production in microglia of the SN (Figure 6A,C,D), compared with shCtrl-injected control rats at 2 weeks post MPP^+^ injection. The CAP-induced reduction of O_2_^−^ production in microglia was significantly decreased in the SN of MPP^+^-lesioned rats treated with CNTFRαNAb compared with non-specific IgG as a control (Figure 6A,C,D). Taken together, these results indicate that CNTF derived from TRPV1 activated astrocytes inhibits microglial ROS production through CNTFRα on microglia in the SN of MPP^+^-lesioned rat.

## 3. Discussion

**Figure 7:**
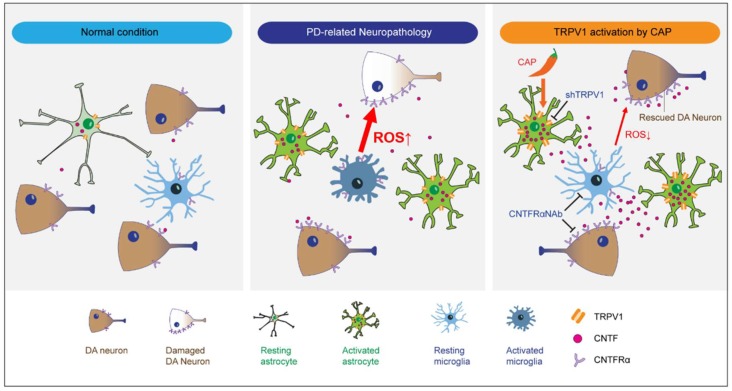
Microglial ROS production is attenuated by astrocytic TRPV1-derived CNTF, resulting in survival of nigral dopamine neurons in Parkinson’s disease. There is a dramatic expression of CNTFRα in reactive microglia in the substantia nigra pars compacta (SNpc) of human Parkinson’s disease and Parkinson’s disease-related neuropathology, compared to the respective controls. Note that astrocytic TRPV1-derived CNTF by CAP inhibits microglial ROS production through CNTFRα on microglia and rescues dopamine neurons in the SNpc of Parkinson’s disease in vivo. TRPV1, Transient Receptor Potential Vanilloid 1; shTRPV1, TRPV1 shRNA lentivirus; DA, Dopamine; CNTF, Ciliary Neurotrophic Factor; CNTFRα, CNTF receptor alpha; CNTFRαNAb, CNTF receptor alpha neutralizing antibody; CAP, capsaicin.

The present study demonstrates that CNTF originated from TRPV1 activated astrocytes inhibits microglial activation and activated microglia-derived oxidative stress through CNTFRα on microglia and rescues DA neurons in the SN of MPP^+^-lesioned rats, an animal model of PD (Figure 7). Intriguingly, compared to control, microglia show no change in expression of endogenous CNTF with a significant increase in CNTFRα in MPP^+^-lesioned rat SN in vivo. Immunohistochemical analysis revealed a significant increase in levels of CNTFRα expression on microglia in postmortem SN tissue from humans with PD, indicating that these findings may have relevance to this disease.

Many studies on PD pathology suggest that glial activation along with neuro-inflammatory processes, like microglial activation, astrogliosis and infiltrated immune cells, contribute to the initiation or progression of PD [38]. DA neurons in the SN are particularly susceptible to oxidative stress because of severe depletion of antioxidants by accumulation of iron and ageing [40,41,42]. Activated microglia generates ROS, which may impose oxidative stress on DA neurons in the SN of both patients with PD [3,4,7] and MPP^+^-lesioned rats [9], leading to DA neuronal cell death. We recently reported that CAP contributes to DA neuronal survival by inhibiting microglia-derived oxidative stress in the SN of MPP^+^-lesioned rats [9]. This study also showed that CAP neuro-protection was masked by pharmacological inhibition of TRPV1 with capsazepine, an antagonist of TRPV1, suggesting TRPV1 involvement. This hypothesis was further confirmed and strongly supported by the present data showing that the CAP-induced neuro-protection and behavioral recovery attained through inhibiting microglia-derived oxidative stress are abolished by genetic knockdown of TRPV1 with shTRPV1 [13]. Consistent with our recent report [13], the present study demonstrated selective inhibition of astrocytic TRPV1 function by shTRPV1, indicating that astrocytic TRPV1 activation, in particular, contributes to inhibiting microglia-derived oxidative stress in the SN of MPP^+^-lesioned rats.

CNTF, expressed in astrocytes upon brain injury [21,43,44] has neuro-protective properties [37], inhibiting microglial activation [29], microglia-derived oxidative stress [25] or both. Consistent with our recent data [9,13], CNTF was expressed on astrocytes in the SN of MPP^+^-lesioned rats and treatment with CNTFRαNAb reduced CAP-induced neuro-protection. Accompanying the lack of neuro-protection was a reduction of CAP effects on microglia-derived oxidative stress, suggesting the inhibitory effects of astrocytic CNTF on microglial activation.

Although our data point to the beneficial effects of CNTF, originating from astrocytes, in particular, the possibility remains that CAP-induced TRPV1 activation produces microglia-originated CNTF, resulting in neuro-protection and inhibition of microglial activation-mediated oxidative stress. This hypothesis is supported by several lines of evidence showing that TRPV1 is expressed on microglia [45], particularly in the SN of MPP^+^-lesioned rats [13] and that microglia produces CNTF following several types of stimulation, including light-induced retinal degeneration [22] and cuprizone-induced demyelination [23]. However, our immunohistochemical analysis revealed little CNTF expression in the SN microglia of MPP^+^-lesioned rats with or without CAP administration. It is therefore likely that CNTF is primarily expressed in astrocytes, but not in microglia under our experimental conditions.

Although CNTF appeared to exert neuro-protection via CNTFRα located on DA neurons [13], several in vitro studies have shown CNTFRα expression on microglia [25,33]. The present study demonstrated a substantial expression of CNTFRα on microglia in the SN of MPP^+^-lesioned rats and a CNTFRαNAb-induced reduction in CAP effects, suggesting that CNTFRα located on microglia could be involved in neuronal survival through inhibition of microglial activation. This microglial CNTFRα-mediated effect would be in addition or parallel to CNTF actions on DA neurons. This observation appears to be important under pathological conditions, given that microglia-derived oxidative stress plays a pivotal role in the initiation of PD, its progression, or both. Therefore, it is likely that CNTF derived from CAP-induced astrocytic TRPV1 activation can act as an endogenous neurotrophic factor, contributing both directly and indirectly to DA neuronal survival in the MPP^+^ rat model of PD.

We have recently shown that CNTFRα was expressed in TH^+^ cells in the SN of intact rat, and CNTFRα expression in TH^+^ cells was significantly decreased in the SN of MPP^+^-lesioned rat without change of total CNTFRα expression [13]. Regarding this, in the present study, the total expression of CNTFRα is not changed, whereas there is significantly increased CNTFRα expression within OX-42^+^ cells in the SN of MPP^+^-lesioned rat. Accordingly, increased level of CNTFRα expression in OX-42^+^ cells may reflect decreased levels of CNTFRα expression in TH^+^ cells in the SN of MPP^+^-lesioned rat.

Taken together with our recent data (TRPV1 and CNTF expression on astrocytes, and CNTFRα expression on DA neurons) [13], CNTFRα expression on microglia in the SN of both humans with PD and MPP^+^-lesioned rats suggests that endogenous CNTF derived from TRPV1 activated astrocytes has therapeutic potential to restore motor function in the treatment of PD by inhibiting activated microglia-mediated oxidative stress and/or preventing the progressive degeneration of DA neurons.

## 4. Materials and Methods

### 4.1. Animals

All experiments were done in accordance with Institutional Animal Care and Use Committee of Kyunghee University and to minimize number of animal experiments and suffering, we carried out the experiment with strict observance of the protocols and guidelines established by Kyung Hee University (KHUASP (SE)-16-059, 8 August 2016). Female Sprague–Dawley rats (10 weeks of age, 240–270 g, purchased from Daehan Biolink, introduced from Taconic Co., Albany, NY, USA) were housed under a 12:12 h light: dark cycle at an ambient temperature of 22 °C. Water and rat chow were available ad libitum.

### 4.2. Human Samples

Human brain tissues (age-matched control and Parkinson’s disease subjects obtained from the Victoria Brain Bank Network (VBBN), Table 1) were prepared to immunohistochemical staining [13]. Human tissue experiments were done in accordance with the Institutional Review Board of Kyung Hee University Hospital (KMC IRB 1234-04, 30 November 2012).

### 4.3. Stereotaxic Surgery and Tissue Preparation

Stereotaxic surgery under chloral hydrate was performed as described [13]. Using coordinates relative to the bregma, stereotaxic injections of MPP^+^ (right medial forebrain bundle (MFB); A/P -3.6, ML -2.0, DV -7.5; MPP^+^, 7.4 μg in 2 μL phosphate-buffered saline (PBS), Sigma, St. Louis, MO, USA), lentivirus (right SN; A/P -5.3, ML -2.3, DV -7.6; shTRPV1, shCtrl; 0.2 μL/min, total 3 μL), CNTFRαNAb (right SN; A/P -5.3, M/L -2.3, D/V -7.6; R&D, AF-303-NA; 0.01 mg/mL, 0.2 μL/min, total 2 μL) and respective control were done according to the atlas of Paxinos and Watson [46]. Lentivirus was injected immediately after MPP^+^ injection. CNTFRαNAb was injected at 1 week post MPP^+^ injection. As described [47], animals were anesthetized with chloral hydrate (360 mg/kg, i.p. injection) at the indicated time points after injection and transcardially perfused and fixed with 4% paraformaldehyde dissolved in 0.1 M phosphate buffer (PB). Brains were removed from the skull, post fixed overnight at 4 °C in buffered 4% paraformaldehyde, and stored at 4 °C in 30% sucrose solution until they sank. Brains were frozen sectioned using a sliding microtome into 40 μm coronal sections and collected in six separate series of SN and striatum.

### 4.4. Capsaicin (CAP) Injection

Before choosing route and dose of CAP injection used here, we measured behavioral changes to avoid painful activation (behavior) of peripheral TRPV1-containing neurons (Table 2). All rats intraperitoneally received a single injection of capsaicin (1, 5, and 10 mg/kg) per day for 7 days. Based on behavioral symptoms and previous results [13,48], CAP (1 mg/kg, i.p. injection; a single injection/day for 7 days, Sigma) was injected at 1 week and 1 day post MPP^+^ injection.

### 4.5. Rotational Behavior Test

As described [13], D-Amphetamine (5 mg/kg, i.p. injection) was used to monitor ipsilateral rotation in rats with unilaterally MPP^+^ administration. Rats that exhibited ipsilateral rotations, indicative of an effective lesion were randomly selected for treatment with the CAP (1 mg/kg, i.p. injection) or vehicle each day for 7 days. The ipsilateral rotations were counted for 1 h. Rats were transcardially perfused for immunostaining at 30 min after the last rotation experiment.

### 4.6. Stereological Estimation

As described [13], the total number of TH^+^ neurons were counted in the various animal groups using the optical fractionator method performed on a bright field microscope (Olympus Optical, BX51, Tokyo, Japan) using Stereo Investigator software (MBF Bioscience, Williston, VT, USA). This unbiased stereological method of cell counting is not affected by either the reference volume SN or the size of the counted elements (neurons). According to Paxinos and Watson [46], all assessments were analyzed in six separated sections in the front and the rear of each target area. As described previously [47], the quantification area for SN are -4.56 mm to -6.60 mm from bregma in the total area.

### 4.7. Morphological Analysis

Optical densities of the TH^+^ striatal fibers were measured using Science Lab 2001 Image Gauge (Fujifilm, Tokyo, Japan) [13]. According to Paxinos and Watson [46], all assessments were analyzed in six separated sections in the front and the rear of each target area. The quantification areas for striatal fibers are +2.16 mm to +0.12 mm from bregma in the total area.

### 4.8. Image J Analysis

Imaging data were analyzed in Image J (National Institutes of Health) as described recently [13]. Image J with co-localization plugin was used to quantify immunofluorescence and with color deconvolution plugin was used to quantify chromogenic signal intensity on image.

### 4.9. Immunostaining

As described [13], rat brain tissues were prepared for immunohistochemical staining. In brief, brain sections (3 tissues per each animal) were rinsed and then incubated with the following primary antibodies: rabbit anti-TRPV1 (1:1000, Alomone labs, Jerusalem, Israel), rabbit anti-CNTF (1:200, Santa-Cruz), goat-anti-CNTFRα (1:200, Santa Cruz, Santa Cruz, CA, USA), mouse and rabbit anti-GFAP (1:500, mouse, Sigma, St. Louis, MO, USA; 1:5000, rabbit, Neuromics, Edina, MS, USA) for astrocytes, rabbit anti-tyrosine hydroxylase (TH, 1:2000, rabbit, Pel-Freez, Rogers, AR, USA) for dopamine neurons and mouse anti-OX-6 (1:400, BD Pharmingen, San Jose, CA, USA), mouse anti-OX-42 (1:400, mouse, BIO-RAD, Hercules, CA, USA) and anti-Iba-1 (1:1000, rabbit, Wako, Richmond, VA, USA) for microglia. Stained tissues were viewed using a confocal microscopy (LSM700, Carl Zeiss, Oberkochen, Germany) or were analyzed under a bright-field microscope (Olympus, Tokyo, Japan). For Human brain experiments, brain sections were deparaffinized, subjected to citrate antigen retrieval prior to immunohistochemistry, washed in cold phosphate-buffered saline (PBS) and block with universal blocking solution at room temperature. Primary antibodies of Iba-1 (1:1000, rabbit, Wako, Richmond, VA, USA), CNTFRα (1:200, goat, Santa Cruz, Santa Cruz, CA, USA) were diluted in 1–5% BSA or normal goat serum and incubated according to manufacturer recommendations. For fluorescent microscopy, brain tissues were labeled using FITC-conjugated-anti-mouse or goat (1:400, Millipore, Burlington, MA, USA), Cy3-conjugated-anti-mouse (1:400, Millipore, Burlington, MA, USA), CF405M-conjugated-anti-mouse IgG (1:400, Biotium, Fremont, CA, USA) and Texas Red-conjugated-anti-rabbit (1:400, Vector Laboratories, Burlingame, CA, USA) secondary antibodies with 4’,6-diamidino-2-phenylindole (DAPI) nuclear counterstain (Vector Laboratories, Burlingame, CA, USA). After washing with PBS, coverslips were mounted on glass slides using mounting media (Vector Laboratories), and analyzed using a confocal microscope (LSM700, Carl Zeiss, Oberkochen, Germany). 

### 4.10. TRPV1 shRNA and Lentivirus Production

As described [13], for plasmid-based short hairpin (sh) RNA expression, the following complementary oligonucleotides were annealed and inserted into the HindIII/BglII sites of pSUPER-EGFP vector: gcgcatcttctacttcaac (sense) TTAGCACTG (loop) gttgaagtagaagatgcgc (antisense), corresponding to nucleotide sequence of TRPV1 [49]. For lentivirus-based shRNA expression, a lentiviral vector containing TRPV1 gene was constructed by inserting synthetic double-strand oligonucleotides 5′-CGCTGCAGTTGCCAACTTGTCAATGAATTCAAGAGATTCATTGACAAGTTGGCAATTTTTGATATCTAGACA-3′ into the HpaI–XhoI restriction enzyme sites of the pSicoR-mcherry lentiviral vector. A shLenti construct containing scrambled oligonucleotides: 5′-CGCATAGCGTATGCCGTTTTCAAGAGAAACGGCATACGCTATGCGATTTTTTC-3′ was used as a control.

### 4.11. In Situ Detection of O_2_^−^ and O_2_^−^-Derived Oxidants

As previously described [9], hydroethidine histochemistry was performed for in situ visualization of O_2_^−^ and O_2_^−^-derived oxidants. At 2 weeks post MPP^+^ injection, hydroethidine (1 mg/mL in PBS containing 1% dimethyl sulfoxide; Sigma) was administered through tail vein. After 45 min, the animals were transcardially perfused, fixed and brain tissues (40 μm thickness) were mounted on gelatin-coated slides. The oxidized hydroethidine product, ethidium, examined by confocal microscopy and merged with OX-42 antibody for double-immunofluorescence staining.

### 4.12. Statistical Analysis

All values are expressed as mean standard error of the mean. Statistical significance (*p* < 0.05 for all analysis) was assessed by One way ANOVA Newman–Keuls analyses and Student unpaired *t*-test (GraphPad Software, San Diego, CA, USA). *F* values were provided in each figure legends.

## Figures and Tables

**Table 1:** Human postmortem tissues used for immunofluorescence in Figure 4D–F.

Sample No.	Final Diagnosis	Age	Sex	PMD	Staining	Tissue
04-424	Control	75.1	F	22.5	Iba-1 + CNTFRα	SN
08-026		67.3	F	24		
07-787		66.5	M	19		
09-260	PD	66.8	F	20		
V11-042		72.1	M	25		
V11-007		74	F	14		

M, Male; F, Female; PMD, Postmortem delays; SN, Substantia nigra.

**Table 2:** Results of capsaicin dose effect test in lethality, pain degree and behavioral symptoms.

Capsaicin Dose (mg/kg)	Lethality			Pain Degree	Behavioral Symptoms (Paroxysm, Spasticity, Couching)
	Dead	Total	%		
1 mg/kg	0	18	0	+	Last for 1–2 min after capsaicin injection only at 1st day
5 mg/kg	1	15	6.6	++	Last for 5–10 min after capsaicin injection for 7 days
10 mg/kg	9	15	60	+++	Last for more than 10 min after capsaicin injection for 7 days Some of rat (*N* = 9) was dead in less than 1 min

+, slight response; ++, medium response; +++, strong response.

## Supplementary Materials

Supplementary materials can be found at http://www.mdpi.com/1422-0067/19/11/3543/s1.
