# Supplementary material for: Inhibition of Microglia-Derived Oxidative Stress by Ciliary Neurotrophic Factor Protects Dopamine Neurons In Vivo from MPP+ Neurotoxicity
Source: Int J Mol Sci. 2018 Nov 10;19(11):3543. doi: 10.3390/ijms19113543 (PMC6274815; doi:10.3390/ijms19113543)
Supplement: Supplementary file 1 [file ijms-19-03543-s001.zip › supplementary.docx]

**
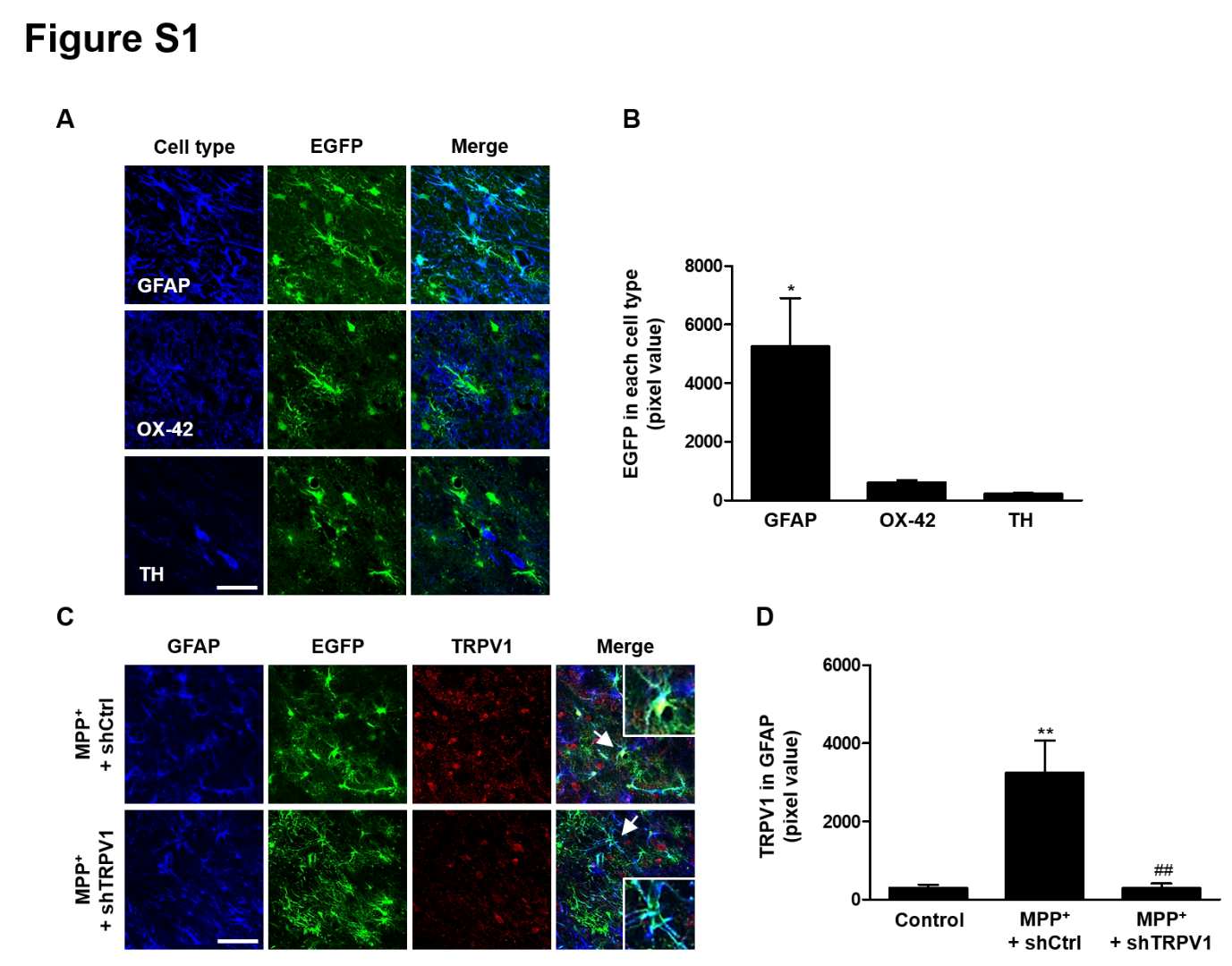
**

**Figure S1.** Specificity of TRPV1 knockdown. MPP^+^ was unilaterally injected into rat MFB followed by injection of shCtrl (control) or shTRPV1 into the SN. At 1 week post MPP^+^, brain tissues were prepared for immunohistochemical analysis. (**A**) Fluorescence images of EGFP (green) or GFAP (blue), OX-42 (blue), TH (blue) and both images are merged in the SN of MPP^+^-lesioned rat. (**B**) Quantification of EGFP expression in each cell type (GFAP^+^, OX-42^+^ and TH^+^). Student t-Test analysis, * *p* <0.05 (t=3.132, df=4), significantly different from control. (**C**) Fluorescence images of GFAP (blue) or EGFP (green), TRPV1 (red), and both images are merged in the SN of MPP^+^-lesioned rat. (**D**) Quantification of TRPV1 expression in GFAP^+^ cell. One way ANOVA [F(2,11)=11.36, P=0.0021] and Newman-Keuls analysis, ^**^ *p* <0.01, significantly different from control. ^##^ *p* < 0.01, significantly different from MPP^+^ + shCtrl. Scale bar; 50 μm (**A** and **C**), Mean ± S.E.M.; B, *n* = 3. D, *n* = 4 to 5
